# Two Luminescent Iridium Complexes with Phosphorous Ligands and Their Photophysical Comparison in Solution, Solid and Electrospun Fibers: Decreased Aggregation-Caused Emission Quenching by Steric Hindrance

**DOI:** 10.3390/ma14185419

**Published:** 2021-09-19

**Authors:** Chaohui Huang, Bin Li

**Affiliations:** 1 School of Materials Science and Engineering, Shandong University, Jinan 250061, China; chaohuipala@163.com; 2 Changchun Institute of Optics Fine Mechanics and Physics, Chinese Academy of Sciences, Changchun 130033, China

**Keywords:** ACQ, Ir complexes, phosphorescence

## Abstract

In this paper, we prepared two phosphorescent Ir complexes with ligands of 2-phenyl pyridine (ppy), and two phosphorous ligands with large steric hindrance, hoping to allow enough time for the transformation of the highly phosphorescent ^3^MLLCT (metal-to-ligand-ligand-charge-transfer) excited state. Their large steric hindrance minimized the π-π interaction between complex molecules, so that the aggregation-induced phosphorescence emission (AIPE) influence could be minimized. Their single crystals indicated that they took a distorted octahedral coordination mode. Photophysical comparison between these Ir complexes in solution, in the solid state and in electrospun fibers was performed to confirm the realization of limited aggregation-caused quenching (ACQ). The potential surface crossing and energy transfer from ^3^ML_BPE_CT/^3^ML_BPE_L_ppy_CT to ^3^ML_ppy_CT in these Ir complexes were revealed by density functional theory calculation and temperature-dependent emission. It was confirmed that these two phosphorous ligands offered large steric hindrance, which decreased the ACQ effect, allowing the efficient emissive decay of the ^3^ML_ppy_CT excited state. This is one of the several luminescent Ir complexes with a high emission yield (Φ = 0.27) and long emission lifetime (0.43 μs) in the solid state.

## 1. Introduction

The development of optoelectronic techniques has attracted research attention for luminescent materials with unique optoelectronic features [1]. Ir complexes have found applications in OLEDs (organic light emitting diodes), solar cells, chemosensing and biosensing, owing to their diverse photophysical features such as their high emission yields, controllable emission color, good thermal stability, long emission lifetime and large Stock’s shift [2,3]. Generally, a luminescent Ir complex is composed of a central Ir(III) ion and three bidentate chelating ligands, forming an octahedral coordination structure. These three bidentate chelating ligands may be homogeneous ones (three C^N chelating ligands) or heterogeneous ones (two C^N and one L^X chelating ligands). Here, C^N is called the major ligand and L^X is called the auxiliary ligand. The photophysical performance of resulting Ir(C^N)_3_ and Ir(C^N)_2_(L^X) complexes is usually dominated and affected by the major ligand, including its molecular structure and conjugation size, while the auxiliary ligand exerts its moderate effect on photophysical performance, so that some desired features can be modified or enhanced, with emission features well preserved [4,5].

Guided by this conclusion, numerous luminescent Ir complexes with molecular formulas of Ir(C^N)_3_ and Ir(C^N)_2_(L^X) have been synthesized and reported. Most of them have shown a high emission yield and shining optoelectronic performance in a highly dispersed state (solution or doped in host) [1,6]. On the other hand, in the solid state or concentrated state, most of these Ir(C^N)_3_ and Ir(C^N)_2_(L^X) complexes face a severe emission quenching effect called aggregation-caused quenching (ACQ) [6]. This effect actually brings a lot of limitations to their practical application. For example, OLEDs using an Ir-based emitting layer have to adjust their doping ratio to balance enough of an emission intensity and limited ACQ effect, which makes the device optimization a complicated task [6]. Tang and coworkers reported on aggregation-induced emission (AIE) firstly in 2001, a study which partially solved the ACQ problem [7]. In 2008, Li and coworkers examined some Ir complexes with strong phosphorescence at room temperature in the solid state, owing to the transformation from a poorly luminescent ^3^LC (ligand centered) to a highly phosphorescent ^3^MLLCT (metal-to-ligand-ligand charge transfer) excited state, which is known as aggregation-induced phosphorescence emission (AIPE) [8]. Nevertheless, AIPE depends on the π-π interaction between complex molecules, which makes the AIPE effect unstable and sensitive to the surrounding environment. In other words, the emission color and quantum yield may be affected by various factors.

**Scheme 1:**
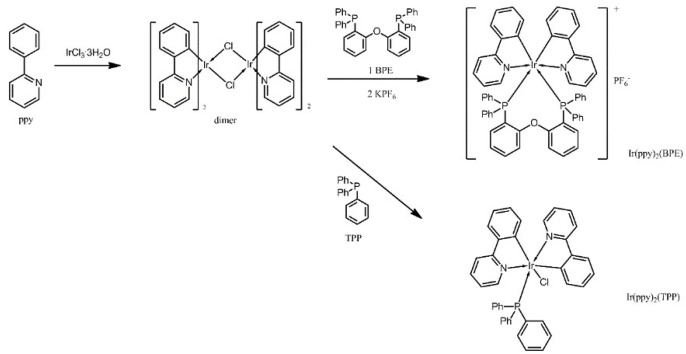
The synthetic route for Ir(ppy)_2_(BPE) and Ir(ppy)_2_(TPP).

Given this fact, luminescent Ir complexes with a low intrinsic ACQ effect should be developed. It has been mentioned above that the emission performance of Ir(C^N)_2_(L^X) complexes is controlled by the C^N ligand, while the L^X ligand serves as an auxiliary one. In this case, we decided to focus on the auxiliary L^X ligand. As shown in Scheme 1, in this work, we selected a classic C^N ligand of 2-phenyl pyridine (ppy) to ensure the emission feature. Two phosphorous ligands with a large steric hindrance were used as the L^X ligand to allow enough time for the transformation to the highly phosphorescent ^3^MLLCT (metal-to-ligand-ligand charge transfer) excited state. Meanwhile, their large steric hindrance may minimize the π-π interaction between complex molecules, so that the AIPE influence can be minimized. Photophysical comparison between these Ir complexes in solution, in the solid state, and in electrospinning fibers was performed to confirm the limited aggregation-caused quenching. This is one of the several luminescent Ir complexes with a high emission yield (Φ = 0.27) in the solid state.

## 2. Materials and Methods

### 2.1. General Information

The synthetic route for the desired Ir complexes, Ir(ppy)_2_(BPE) and Ir(ppy)_2_(TPP), is shown in Scheme 1. The chemicals and regents involved in Scheme 1 were commercially obtained and used as received, including bis(2-(diphenylphosphanyl)phenyl) ether (BPE), triphenylphosphane (TPP), 2-phenyl pyridine (ppy), KPF_6_, PVP (K30) and IrCl_3_·3H_2_O. MS and NMR spectra were recorded using a Bruker Avance 500 spectrometer and an Agilent 1100 MS spectrometer, respectively. Single crystal diffraction data were obtained with a Bruker SMART APEX II X-ray single crystal diffractometer. Density functional theory calculation was finished with GAMESS at the RB3LYP/LANL2DZ level. A single crystal structure was applied as the initial geometry. Absorption spectra were recorded using a Shimadzu UV-3101PC spectrophotometer. Emission spectra, lifetime, and quantum yield were determined using a Hitachi F-7000 fluorescence spectrophotometer equipped as an integrating sphere. Micromorphology was investigated using a Hitachi S-4800 microscope and a Nikon TE2000-U fluorescence microscopy.

### 2.2. Synthesis of Ir(ppy)_2_(BPE) and Ir(ppy)_2_(TPP)

Firstly, Ir dimer was synthesized following a literature method and used as a starting compound [9]. 2-ethoxyethanol (20 mL) containing IrCl_3_·3H_2_O (0.7 mmol) was mixed with water (7 mL) and then heated to reflux under an Ar atmosphere for 25 h. Solid powder was precipitated by adding water (10 mL) into the cooled reaction solution. The resulting solid product (dimer) was collected and mixed with BPE (1.4 mmol). Then, MeOH (5 mL) and CH_2_Cl_2_ (10 mL) were added. The resulting solution was heated to reflux under an Ar atmosphere for 20 h. After its being cooled to room temperature, KPF_6_ (7 mmol) was added and stirred for another 2 h. The resulting solid product was recrystallized in MeOH to give a white solid product as Ir(ppy)_2_(BPE). Yield (47%). ^1^H NMR (DMSO-D_6_) δ 8.85–8.83 (m, 2H), 8.43–8.40 (m, 2H), 8.29–8.27 (m, 2H), 7.82–7.79 (m, 4H), 7.41–7.46 (m, 30H), 7.17–7.13 (m, 4H). ESI-MS: *m*/*z* = 1039.3. Its single crystal will be discussed below.

Ir(ppy)_2_(TPP) was synthesized following a similar procedure, except that BPE was replaced by TPP in this run. The solid product was recrystallized in MeOH to give the white solid product. Yield (51%). ^1^H NMR (DMSO-D_6_) δ 8.86–8.83 (m, 2H), 8.45–8.41 (m, 2H), 8.29–8.26 (m, 2H), 7.83–7.78 (m, 4H), 7.51–7.46 (m, 21H). ESI-MS: *m/z* = 798.2. Its single crystal will be discussed below.

### 2.3. Preparation of Electrospun Fibers Doped with Ir(ppy)_2_(BPE) and Ir(ppy)_2_(TPP)

A brief description for the preparation of electrospun fibers doped with Ir(ppy)_2_(BPE) and Ir(ppy)_2_(TPP) is given as follows. Host PVP was dissolved in DMF and stirred carefully to form a transparent solution, and then dopant (Ir(ppy)_2_(BPE) or Ir(ppy)_2_(TPP)) was added and stirred for another 30 min with a spinning speed of 60 rounds/minute. The resulting solution was transferred into a plastic syringe with a needle and wired to the anode terminal of a high-voltage power supply by a copper wire. A collecting board made of Al foil was wired to the ground electrode so that an electrospinning procedure could be performed.

## 3. Results and Discussion

### 3.1. Characterization of Ir(ppy)_2_(BPE), Ir(ppy)_2_(TPP) and Their Doped Electrospun Fibers

#### 3.1.1. Crystal Structure of Ir(ppy)_2_(BPE) and Ir(ppy)_2_(TPP)

**Figure 1:**
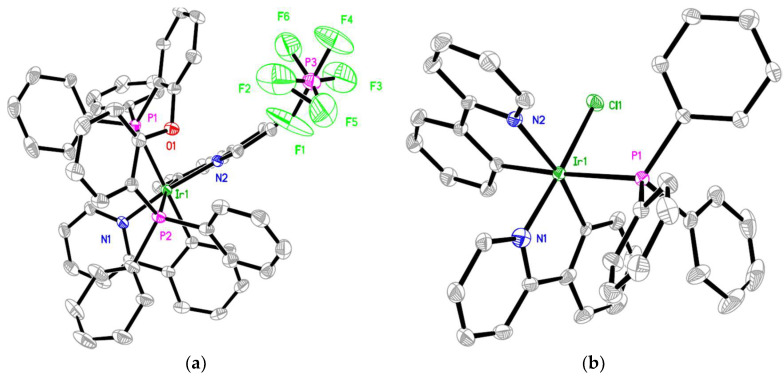
Single crystal structure of Ir(ppy)_2_(BPE) (**a**) and Ir(ppy)_2_(TPP) (**b**).

Single crystals of Ir(ppy)_2_(BPE) and Ir(ppy)_2_(TPP) were obtained. It was found that they tended to crystalize in a monoclinic system, as shown by the selected cell parameters listed in Table 1. A visual understanding of these two single crystals is shown as Figure 1. Like other Ir(III) complexes with ppy as the C^N ligand, Ir(ppy)_2_(BPE) and Ir(ppy)_2_(TPP) adopted an octahedral coordination mode, which was slightly distorted due to their heterogeneous ligands. As for Ir(ppy)_2_(BPE), two ppy ligands and one BPE ligand coordinated with the Ir(III) ion, forming an octahedral sphere. In Ir(ppy)_2_(TPP), two ppy ligands, one TPP ligand and one Cl atom led to an octahedral sphere as well. Here, the Cl atom served as a counterion and a ligand at the same time. The steric hindrance between the TPP molecules prohibited a second TPP from coordinating with the Ir(III) ion. From Table 1, it is observed that Ir-N and Ir-C bonds of Ir(ppy)_2_(BPE) were longer than the corresponding values of Ir(ppy)_2_(TPP). These values were even larger than the literature values of other bis-cyclometalated complexes with acetylacetone as the L^X ligand (<2.0 Å) [10,11]. This fact suggests that BPE and TPP with a large steric hindrance partially weaken the coordination affinity of the ppy ligand.

Although the Cl atom is small enough to avoid the effect from the TPP steric hindrance, the Ir-Cl bond in Ir(ppy)_2_(TPP) was still longer than the literature values [10,11]. In addition, the Ir-P bond length values in Ir(ppy)_2_(BPE) and Ir(ppy)_2_(TPP) (~2.5 Å) were longer than those in other metal complexes with ligands of BPE and TPP (<2.3 Å) [10,11]. This fact indicates that the large steric hindrance of the BPE and TPP ligands weakens their own coordination affinity as well. As we mentioned above, such a large steric hindrance may improve the emissive performance of Ir(ppy)_2_(BPE) and Ir(ppy)_2_(TPP) in the solid state by reducing the ACQ effect, which will be discussed below. These bond length and bond angle values of Ir(ppy)_2_(BPE) and Ir(ppy)_2_(TPP) were generally rather similar to the literature values of Ir(III) single crystals based on similar ligands (CCDC 265264, 821321, 854016, 952679, 1423257 and 1576156) [12,13,14,15,16]. The Ir-N bond length values of Ir(ppy)_2_(BPE) and Ir(ppy)_2_(TPP) were slightly longer than the equivalent literature values (by ~0.2 Å), but their bond angles were much similar to the literature values. This result suggests that the BPE and TPP ligands try to decrease the steric hindrance around the Ir(III) center by extending a coordination bond, instead of distorting the coordination field.

Despite the obvious effect on the bond length, the steric hindrance of the BPE and TPP ligands exerted no strong effect on the bond angle. For example, all N-Ir-C bond angles were close to 80° and comparable to the literature values of ~79 ° with small ligands [10,11]. The P-Ir-P bite angle of Ir(ppy)_2_(BPE) was as large as 99° and comparable to the natural bond angle of the BPE ligand (102°) [17]. This fact suggests that the BPE ligand and TPP ligand distort their structures to meet the optimal coordination angle with the central Ir(III) ion. This statement is supported by the long distance between the Ir and the O atom from the BPE ligand (~3.5 Å), which is even longer than the literature values.

Meanwhile, the large steric hindrance made the Ir(ppy)_2_(BPE) and Ir(ppy)_2_(TPP) molecules disperse in the crystal state, showing no valid π-π interaction, as shown in Figure 1. The average distance between two neighboring Ir(III) ions was determined as 9.78 Å for Ir(ppy)_2_(BPE), and 9.88 Å for Ir(ppy)_2_(TPP). This long distance may avoid the interaction and corresponding energy annihilation between excited Ir(III) molecules, decreasing the ACQ effect and thus enhancing emissive performance. This hypothesis will be further discussed below.

#### 3.1.2. SEM and Fluorescence Microscope Images of Ir(ppy)_2_(BPE)- and Ir(ppy)_2_(TPP)-Doped Electrospun Fibers

**Figure 2:**
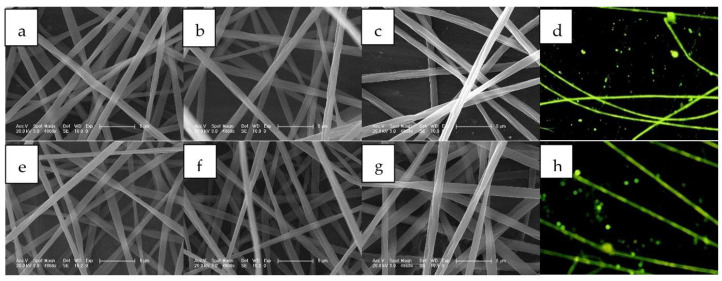
SEM and fluorescence microscope images of Ir(ppy)_2_(BPE) and Ir(ppy)_2_(TPP) in PVP fiber. (**a**) SEM of Ir(ppy)_2_(BPE) in PVP (5%); (**b**) SEM of Ir(ppy)_2_(BPE) in PVP (10%); (**c**) SEM of Ir(ppy)_2_(BPE) in PVP (15%); (**d**) fluorescence microscope image of Ir(ppy)_2_(BPE) in PVP (10%); (**e**) SEM of Ir(ppy)_2_(TPP) in PVP (5%); (**f**) SEM of Ir(ppy)_2_(TPP) in PVP (10%); (**g**) SEM of Ir(ppy)_2_(TPP) in PVP (15%); (**h**) fluorescence microscope image of Ir(ppy)_2_(TPP) in PVP (10%).

Generally, most Ir(C^N)_3_ and Ir(C^N)_2_(L^X) complexes have shown high emission yields and shining optoelectronic performance in a highly dispersed state (solution or doped in a host) [1,6]. Particularly, solid hosts, such as polymer, provide a rigid microenvironment for emitters and restrict excited state relaxation, leading to even better emissive performance than that in solution. In this work, we compared the emissive performance of Ir(ppy)_2_(BPE) and Ir(ppy)_2_(TPP) in solution, in the solid state, and in polymer fiber. Their SEM and fluorescence microscope images in PVP fiber are shown in Figure 2. There were three doping concentrations for each Ir complex: 5%, 10%, and 15%, respectively. When the doping concentration was as low as 5%, a smooth surface was observed for both Ir(ppy)_2_(BPE)- and Ir(ppy)_2_(TPP)-doped fibers, with an average diameter of 1 μm recorded. There was no obvious difference between these two fibers, indicating that the dopants (Ir(ppy)_2_(BPE) and Ir(ppy)_2_(TPP)) had a slim effect on fiber micromorphology, due to the low doping concentration. Upon increasing the doping concentration, the doped fiber tended to show a rough surface, although the mean diameter was preserved as 1 μm. It seemed that a high doping concentration compromised the PVP homogeneity. This is because the PVP host is an organic polymer matrix with low dipole moment, while Ir(ppy)_2_(BPE) and Ir(ppy)_2_(TPP) are metal complexes with counterions. Their high dipole moment makes them incompatible with a PVP host. Nevertheless, there is no phase separation in these doped fibers, indicating that Ir(ppy)_2_(BPE) and Ir(ppy)_2_(TPP) molecules were trapped in the PVP framework.

The successful doping of the Ir(ppy)_2_(BPE) and Ir(ppy)_2_(TPP) molecules in the PVP host can be tentatively confirmed by the fluorescence microscope images shown in Figure 2. Bright green emission was observed from the Ir(ppy)_2_(BPE)-doped fiber, originating from each individual fiber, which means there was a homogeneous distribution of Ir(ppy)_2_(BPE) in the PVP host. As for the Ir(ppy)_2_(TPP)-doped fiber, its emission was much weaker compared to the Ir(ppy)_2_(BPE)-doped fiber. Since there is only one ligand with a high steric hindrance in Ir(ppy)_2_(TPP), it is assumed that this weak emission was caused by the insufficient steric hindrance. In other words, the ACQ effect was still obvious due to the insufficient steric hindrance in Ir(ppy)_2_(TPP). A detailed discussion will be presented below. On the other hand, there was still no phase separation in the Ir(ppy)_2_(TPP)-doped fiber, confirming the successful electrospinning synthesis.

### 3.2. Photophysical Comparison of Ir(ppy)_2_(BPE) and Ir(ppy)_2_(TPP) in Solution, the Solid State, and Electrospun Fibers

#### 3.2.1. Absorption and Excitation Spectra

**Figure 3:**
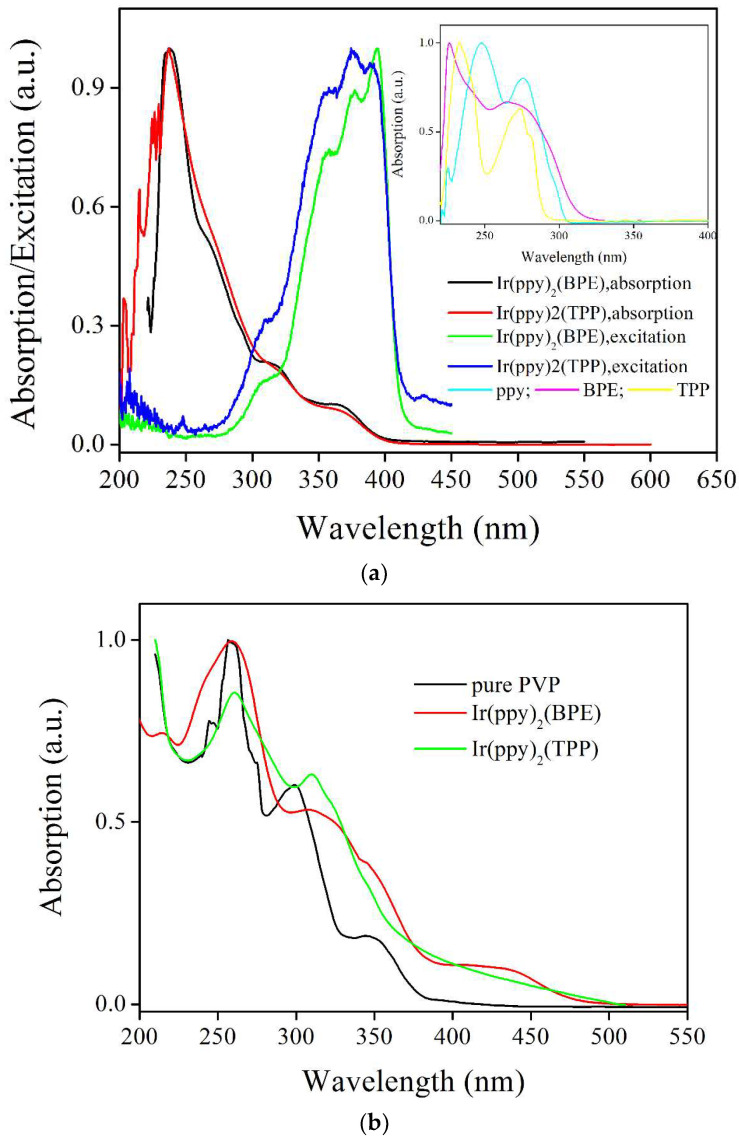
(**a**) Absorption and excitation spectra of Ir(ppy)_2_(BPE) and Ir(ppy)_2_(TPP) in CH_2_Cl_2_ solution (1 μM). Inset: absorption spectra of ligands in CH_2_Cl_2_ solution (1 μM); (**b**) Absorption spectra of pure PVP, Ir(ppy)_2_(BPE)- and Ir(ppy)_2_(TPP)-doped PVP fiber (10%).

The absorption spectra of Ir(ppy)_2_(BPE) and Ir(ppy)_2_(TPP) in CH_2_Cl_2_ solution (1 μM) are recorded and shown in Figure 3. Their detailed photophysical parameters are listed in Table 2, including the absorption peak (λ_abs_), absorption edge (λ_edg_), emission peak (λ_em_), emission lifetime (τ), and emission quantum yield (Φ). Owing to the rather similar molecular structures of Ir(ppy)_2_(BPE) and Ir(ppy)_2_(TPP), their absorption spectra were nearly identical to each other, showing a major band peaking around 237 nm and two shoulder bands peaking at ~318 nm and ~369 nm, respectively. This observation is consistent with the literature conclusion that the photophysical performance of the Ir(C^N)_2_(L^X) complexes is controlled by the C^N ligand, while the L^X ligand serves as an auxiliary ligand which does not noticeably affect photophysical performance [4,5]. On the other hand, a slimmer absorption red shift was observed for Ir(ppy)_2_(BPE) compared to the Ir(ppy)_2_(TPP) absorption red shift. This was because there is an electron-donating O atom in the BPE ligand, which decreases the electronic transition energy of Ir(ppy)_2_(BPE). Their absorption spectra extended to a visible region and ended at ~410 nm, which was obviously red shifted compared to the ligand absorption spectra of ppy, BPE and TPP (ending at ~308 nm for ppy, ~327 nm for BPE, and ~300 nm for TPP, respectively), as shown in the inset of Figure 3. After comparing the absorption spectra of Ir(ppy)_2_(BPE) and Ir(ppy)_2_(TPP) with those of their ligands, their major absorption band at 237 nm was attributed to the π-π* transition of their ligands. The shoulder peak, meanwhile, at 369 nm is a newly generated one and was assigned as the transition of MLLCT, which was confirmed below by theoretical calculation [5].

Regardless of the weak absorption of the MLLCT transition (369 nm), it was highly effective in exciting the emissive centers of Ir(ppy)_2_(BPE) and Ir(ppy)_2_(TPP), as shown in Figure 3, while the strong absorption of the π-π* transition was ineffective for this. This is because the π-π* transition is a spin-allowed transition and thus has a high molar extinction coefficient and strong absorption, while the MLLCT transition is a partially prohibited transition, leading to a low molar extinction coefficient and weak absorption [18]. The emissive center is usually derived from the onset electronic state of MLLCT. In this case, the π-π* excited state had to experience an energy-exhausting procedure of potential surface crossing before transferring its energy to MLLCT, leading to its low excitation efficiency.

After being doped into the PVP fiber, all of the abovementioned absorption bands were well preserved, as shown in Figure 3, indicating the successful preparation of Ir(ppy)_2_(BPE)- and Ir(ppy)_2_(TPP)-doped PVP fibers. It was observed that the absorption spectra of the Ir(ppy)_2_(BPE)- and Ir(ppy)_2_(TPP)-doped PVP fibers were basically the absorption adduct of the Ir complex dopant and PVP host. There was no new absorption band or fine vibrational structures, indicating that there was no strong interaction between the Ir complex dopant and PVP host. In other words, Ir(ppy)_2_(BPE) and Ir(ppy)_2_(TPP) molecules were merely immobilized in the PVP matrix, resulting in a “solid solution”. Such solid solutions have been proven as a perfect medium for emitters, showing stable emission and restricted geometric relaxation of excited states [19]. On the other hand, an obvious red shift was observed for the Ir(ppy)_2_(BPE)- and Ir(ppy)_2_(TPP)-doped PVP fibers, compared to the absorption spectra of Ir(ppy)_2_(BPE) and Ir(ppy)_2_(TPP). Especially, the absorption edge was red shifted by ~100 nm, ending at ~510 nm for both the Ir(ppy)_2_(BPE)- and Ir(ppy)_2_(TPP)-doped PVP fibers. A similar case has been observed for other PVP-based solid solutions [19,20]. A possible explanation is raised as follows. In PVP hosts, dopant molecules are perfectly dispersed and surrounded by a PVP framework. In this case, dopant molecules could be well stabilized in their excited state, leading to decreased transition energy. In addition, the light scattering effect on the PVP fiber surface may shift the absorption edge to a long wavelength as well [20].

#### 3.2.2. Emission Spectra

**Figure 4:**
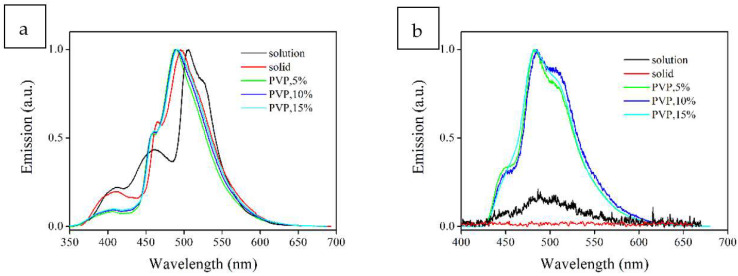
Emission spectra of Ir(ppy)_2_(BPE) (**a**) and Ir(ppy)_2_(TPP) (**b**) in CH_2_Cl_2_ solution (1 μM), in the solid state and in PVP fiber (5%, 10% and 15%).

The emission spectra of Ir(ppy)_2_(BPE) and Ir(ppy)_2_(TPP) in CH_2_Cl_2_ solution (1 μM), in the solid state and in PVP fiber (5%, 10% and 15%) are recorded and shown in Figure 4. Ir(ppy)_2_(BPE) and Ir(ppy)_2_(TPP) presented multiple emission peaks, showing a green emission color. Their detailed emission peaks are listed in Table 2. Generally speaking, the Ir(ppy)_2_(BPE) and Ir(ppy)_2_(TPP) emission energy was higher than that of most Ir(ppy)_2_(L^X) complexes (λ_em_ > 550 nm) [10,19,20]. Since these Ir complexes have the same major ligand, the increased emissive energy is attributed to the BPE and TPP auxiliary ligands, owing to their wide energy band and large steric hindrance, which limits the energy-exhausting procedure of geometric relaxation in the excited state. On the other hand, owing to the electron-donating effect of the O atom and Cl atom in Ir(ppy)_2_(BPE) and Ir(ppy)_2_(TPP), their emission was red shifted (by ~30 nm) compared to similar Ir(ppy) complexes with phosphorous ligands [12,13,14,15,16].

As for Ir(ppy)_2_(BPE), in CH_2_Cl_2_ solution, there were three emission bands, two weak ones peaking at 411 nm and 460 nm, and a major one peaking at 506 nm. There was still a shoulder peak at around 525 nm. The latter two emission peaks are commonly observed for most Ir(ppy)_2_(L^X) complexes, which have been assigned as emission from the ppy-based ^3^ML_ppy_CT excited state [21]. It is clear that the major emission band was well preserved and suffered from a slim effect from the auxiliary ligand. This finding is consistent with a previous literature report [20]. The former two emission bands, however, were newly generated ones. No similar case has been reported for other Ir(ppy)_2_(L^X) complexes. It is thus assumed that the auxiliary ligand (BPE) was involved in the Ir(ppy)_2_(BPE) emitting procedure. Correspondingly, the former two emission bands were tentatively assigned as ^3^ML_BPE_CT (411 nm) and ^3^ML_BPE_L_ppy_CT (460 nm), respectively. This statement was strengthened by theoretical calculation results, which will be discussed below. In the solid state, Ir(ppy)_2_(BPE)’s first emission band was well preserved, but the second and the last shoulder emission bands were weakened, and merged with the major emission band, showing a widened emission band peaking at 466 nm. An emission blue shift of 6 nm was observed, compared to the Ir(ppy)_2_(BPE) emission in solution (460 nm). This is because each Ir(ppy)_2_(BPE) molecule in the solid state is surrounded by neighboring Ir(ppy)_2_(BPE) molecules with strong dipole moment. In this case, the ^3^ML_ppy_CT excited state interacted with this high dipole moment microenvironment, leading to an emission blue shift of the ^3^ML_ppy_CT transition and thus the merging of the latter three emission bands. It is still observed that the first emission band ^3^ML_BPE_CT (411 nm) was well preserved. This is because the BPE ligand has large steric hindrance and partially shields the ^3^ML_BPE_CT excited state from the effects of the surrounding environment. The PVP fiber case was similar to the solid state emission of Ir(ppy)_2_(BPE), with the latter three emission bands merging into a major one, which peaked at ~490 nm. After increasing the doping concentration from 5% to 15%, the major emission band red shifted from 488 nm to 491 nm, indicating that Ir(ppy)_2_(BPE) aggregation became increasingly severe, and close to the solid state case. In addition, the relative emission intensity of the ^3^ML_BPE_CT transition was obviously weakened, indicating a more efficient energy transfer from ^3^ML_BPE_CT to ^3^ML_ppy_CT. This was because the rigid PVP framework and large steric hindrance of the BPE ligand ensure enough time for ^3^ML_BPE_CT to finish this energy transfer.

As for Ir(ppy)_2_(TPP), due to the deficient steric hindrance of the TPP ligand, only a weak emission band was observed in solution, and there was no valid emission signal in the solid state. This fact suggests that without the help of additional steric hindrance, Ir(ppy)_2_(TPP) suffers from intense geometric relaxation in the excited state, leading to the ACQ effect. After being doped into the PVP fiber, Ir(ppy)_2_(TPP) molecules were immobilized in the PVP framework. With this additional steric hindrance, emission was observed for the Ir(ppy)_2_(TPP)-doped fiber, as shown in Figure 4. There were still three emission bands, peaking at ~450 nm, ~485 nm and ~510 nm, respectively, which were assigned as ^3^ML_TPP_L_ppy_CT and ^3^ML_ppy_CT transitions, respectively. Their emissive energy values, however, were noticeably smaller than those of Ir(ppy)_2_(BPE). Considering the similar optical edge values of Ir(ppy)_2_(BPE) and Ir(ppy)_2_(TPP) (in solution, 404 nm vs. 409 nm; in PVP, 510 nm vs. 512 nm), the red shifted emission of Ir(ppy)_2_(TPP)-doped fiber can be attributed to the deficient steric hindrance of TPP, which fails to limit the geometric relaxation of the excited state.

#### 3.2.3. Emission Lifetime and Quantum Yield

**Figure 5:**
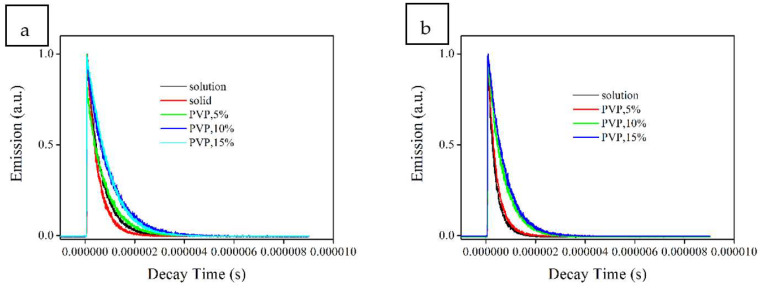
Emission decay dynamics of Ir(ppy)_2_(BPE) (**a**) and Ir(ppy)_2_(TPP) (**b**) in CH_2_Cl_2_ solution (1 μM), in the solid state and in PVP fiber (5%, 10% and 15%).

To confirm the phosphorescence nature of Ir(ppy)_2_(BPE) and Ir(ppy)_2_(TPP), their emission decay dynamics were determined, and these are shown as Figure 5. Detailed lifetime values are listed in Table 2. It was observed that Ir(ppy)_2_(BPE) and Ir(ppy)_2_(TPP) followed the triexponential decay mode in solution, showing three emission decay components. The observation of these three emission decay components suggests that there were at least three emissive decay paths for the Ir(ppy)_2_(BPE) and Ir(ppy)_2_(TPP) excited states. This finding is consistent with the above observation of multiple emission bands from Ir(ppy)_2_(BPE) and Ir(ppy)_2_(TPP), including ^3^ML_BPE_CT (411 nm), ^3^ML_BPE_L_ppy_CT, ^3^ML_TPP_L_ppy_CT and ^3^ML_ppy_CT. Especially, τ_1_ of Ir(ppy)_2_(TPP) was as short as 0.02 μs, which was tenfold smaller than the other two emission decay components, suggesting that there was an energy-competing procedure between these emission decay components via potential surface crossing [21]. Nearly all emission decay components were on the scale of microseconds, indicating their phosphorescent nature. Their weighted mean lifetime values (τ) were calculated as 0.57 μs for Ir(ppy)_2_(BPE) and 0.29 μs for Ir(ppy)_2_(TPP). These values were obviously longer than those of typical Ir(III) complexes (<0.1 μs), which was attributed to the phosphorescent nature of the emissive center, along with the steric hindrance effect of the BPE and TPP ligands [10,12,13,14,15,16]. The shorter lifetime value of Ir(ppy)_2_(TPP) compared to that of Ir(ppy)_2_(BPE) was still attributed to the deficient steric hindrance of the TPP ligand. In the solid state, with increasing interactions between Ir complex molecules, Ir(ppy)_2_(TPP) suffered from an intense ACQ effect and showed no valid emission decay signal. Similarly, the Ir(ppy)_2_(BPE) lifetime decreased to 0.43 μs as well. Its three emission decay components were still observed. Two of them noticeably decreased, while the shortest component τ_1_ remained constant. It was thus further confirmed that there was an energy transfer procedure between these three emission decay components, and τ_1_ was literally controlled by this energy transfer procedure, instead of by the surrounding environment or its non-radiative decay. After being doped into the PVP fiber, only the monoexpoential decay mode was observed, although there were still multiple emission bands. This was because the shoulder emission bands were weak, and their decay behavior was covered up by the major emission band. These emission lifetime values were much longer than those in solution or in the solid state, which means that the excited states of Ir(ppy)_2_(BPE) and Ir(ppy)_2_(TPP) were isolated from the influence of non-radiative decay paths, such as geometric relaxation. This statement will be strengthened below. In addition, it seemed that there was an optimal doping concentration for each dopant. For example, the Ir(ppy)_2_(BPE) emission decay lifetime increased from 0.52 μs to 0.55 μs when the doping concentration increased from 5% to 10%; additionally, by further increasing the doping concentration to 15%, its lifetime decreased to 0.53 μs. This result actually indicates an increasing Ir(ppy)_2_(BPE) aggregation and corresponding ACQ effect, which is consistent with the finding in Section 3.2.2.

To confirm this tendency, the emission quantum yield (Φ) of these samples were determined, and these are listed in Table 2. In solution, Ir(ppy)_2_(BPE) showed a high Φ value of 0.43, while the Ir(ppy)_2_(TPP) Φ value was only as low as 0.01. Considering their rather similar molecular structures, the deficient steric hindrance of the TPP ligand should have been the only reason for the difference between the Ir(ppy)_2_(BPE) and Ir(ppy)_2_(TPP) quantum yields. No valid Φ value was observed for Ir(ppy)_2_(TPP) in the solid state, indicating its intense ACQ effect. Although the Ir(ppy)_2_(BPE) Φ value slightly decreased to 0.27 in the solid state, this value was still comparable to literature values of phosphorescent Ir complexes [10,22]. This was one of the several phosphorescence examples from the solid state Ir complexes [22]. After being doped into the PVP fiber, Ir(ppy)_2_(BPE) and Ir(ppy)_2_(TPP) molecules were immobilized in the PVP framework. With the help of this additional steric hindrance, the ACQ effect and geometric relaxation were well-managed, and their Φ values noticeably increased, as shown in Table 2. Especially, the Φ value of the Ir(ppy)_2_(TPP)-doped fiber was eightfold higher than that of the Ir(ppy)_2_(TPP) solution.

Generally, for a simple emissive center with only two decay paths of radiative decay and non-radiative decay, their decay constants (K_r_ and K_nr_) can be calculated with Formula 1 and Formula 2 [18]. These two formulas, however, are not applicable for Ir(ppy)_2_(BPE) and Ir(ppy)_2_(TPP), since they have multiple emissive decay paths and thus there is energy competition and energy transfer between these procedures. Nevertheless, a tentative discussion can still be made using weighted mean lifetime (τ) and emission quantum yield values.

τ = 1/(K_r_ + K_nr_) (1)

Φ = (K_r_/K_r_ + K_nr_) (2)

It is observed from Table 2 that the K_r_ values of Ir(ppy)_2_(BPE) were similar to each other in various states, falling in a narrow region of 6.28–7.54 × 10^5^ s^−1^. On the other hand, the Ir(ppy)_2_(BPE) K_nr_ value noticeably depended on its state. In the solid state, its K_nr_ value was as high as 16.98 × 10^5^ s^−1^. In solution, this K_nr_ value decreased to 10.01 × 10^5^ s^−1^, and in PVP fiber, due to the immobilization effect of the host framework, the K_nr_ value further decreased to 5.17 × 10^5^ s^−1^. As for Ir(ppy)_2_(TPP), since the TPP steric hindrance is smaller than that of BPE, all its K_nr_ values were much higher than the corresponding values of Ir(ppy)_2_(BPE). For example, in the solid state, the K_nr_ value of Ir(ppy)_2_(TPP) was threefold higher than that of Ir(ppy)_2_(BPE). Although the K_nr_ values of Ir(ppy)_2_(TPP) decreased after being doped into the PVP fiber, they were still two times higher than those of Ir(ppy)_2_(BPE). The systematical comparison between these K_r_/K_nr_ values finally confirmed that the ACQ effect effectively decreased in Ir(ppy)_2_(BPE), showing efficient emission in the solid state.

### 3.3. Confirmation of the Reduced Aggregation-Caused Quenching Effect: Mechanism Analysis

#### 3.3.1. Eliminate the Possibility of the AIPE Effect

**Figure 6:**
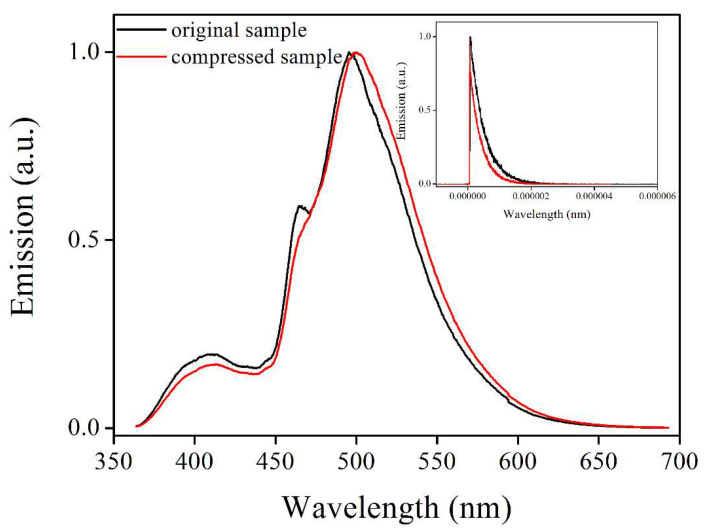
Emission spectra of Ir(ppy)_2_(BPE) solid before and after its being treated with a pressure of 1 MPa. Inset: emission decay dynamics of Ir(ppy)_2_(BPE) solid before and after its being treated with a pressure of 1 MPa.

**Figure 7:**
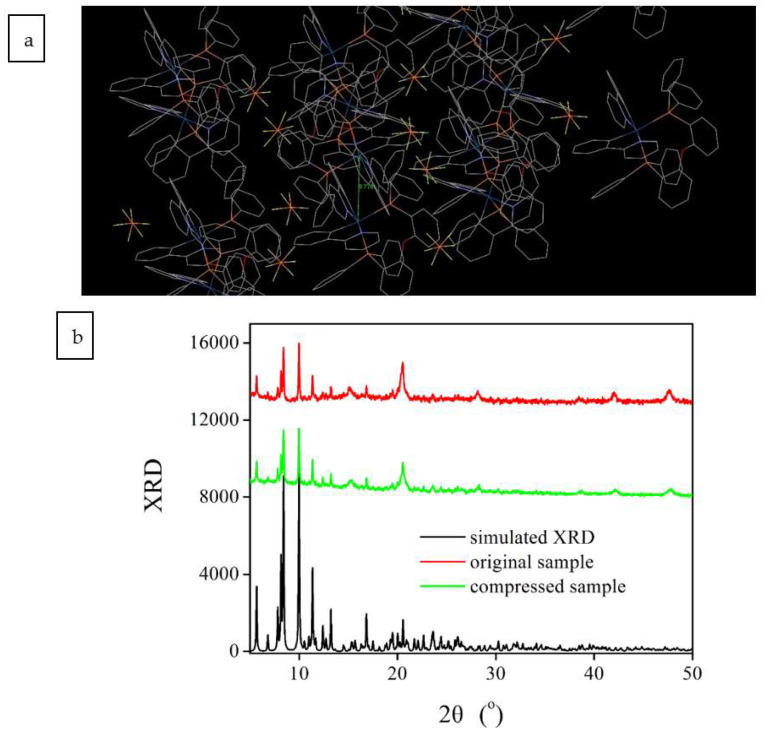
Ir(ppy)_2_(BPE) crystal packing mode (**a**), and XRD patterns of original and compressed Ir(ppy)_2_(BPE) (**b**).

Although the discussions in Section 3.2.2 and Section 3.2.3 suggest that the reduced ACQ effect in Ir(ppy)_2_(BPE) was attributed to its auxiliary BPE ligand and its large steric hindrance, there is still one factor to be considered, which is AIPE (aggregation-induced phosphorescence emission). According to a literature report, AIPE is accomplished by the intermolecular π-π effect, forming a rigid microenvironment that accelerates emissive decay [8]. To eliminate the possibility of the AIPE effect in Ir(ppy)_2_(BPE), XRD patterns and emission spectra of Ir(ppy)_2_(BPE) solid were recoded before and after their being treated with a pressure of 1 MPa. As shown in Figure 6, the compressed Ir(ppy)_2_(BPE) still showed three emission bands, peaking at 410 nm, 465 nm and 501 nm, respectively. The first two emission bands were weakened compared to those of the original Ir(ppy)_2_(BPE), but no emission spectral shift was observed. The latter emission band, however, showed an emission red shift of ~5 nm. This fact suggests that the energy transfer from ^3^ML_BPE_CT (411 nm)/^3^ML_BPE_L_ppy_CT (465 nm) to ^3^ML_ppy_CT (501 nm) became more efficient in concentrated Ir(ppy)_2_(BPE) molecules, owing to the increased environmental polarity in the condensed state. Correspondingly, the emission lifetime of the concentrated Ir(ppy)_2_(BPE) was degraded to the mononexponential mode, compared to the triexponential mode of the original Ir(ppy)_2_(BPE). However, the lifetime and emission yield decreased to 0.36 μs and 0.21, respectively, compared to those of the original Ir(ppy)_2_(BPE). This result actually eliminates the possibility of the AIPE effect in Ir(ppy)_2_(BPE) molecules. A possible explanation for the missing AIPE effect is given as follows. BPE has large steric hindrance and thus makes Ir(ppy)_2_(BPE) molecules disperse, which compromises AIPE formation. To confirm this statement, the stacking mode of Ir(ppy)_2_(BPE) crystal was plotted, and this is presented in Figure 7. No close contact or π-π stacking was observed between neighboring Ir(ppy)_2_(BPE) molecules. Its simulated XRD pattern is shown in Figure 7 and compared to the XRD patterns of the original and compressed Ir(ppy)_2_(BPE). These three curves showed similar peaks within the 2θ region of 5–20°. It was thus confirmed that there was no valid π-π stacking in both the original and compared Ir(ppy)_2_(BPE) samples. The possibility of AIPE can hereby be ruled out.

#### 3.3.2. Analyze the Multiple Emissive Centers via DFT Calculation

**Figure 8:**
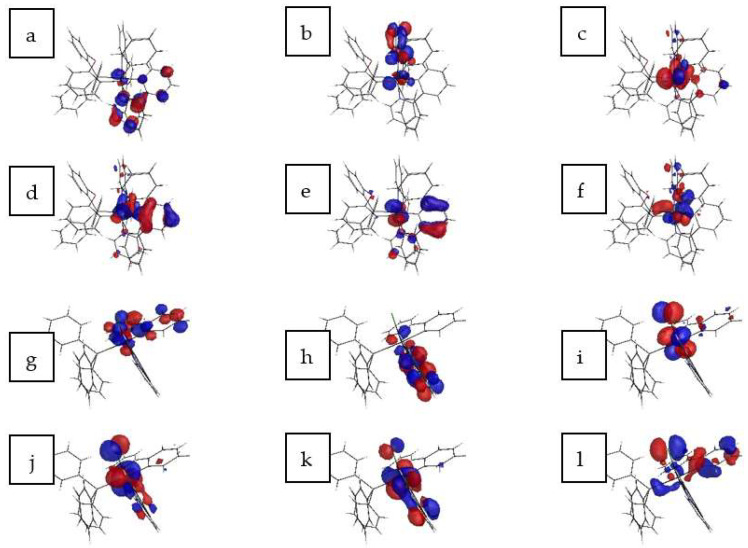
Frontier molecular orbitals of Ir(ppy)_2_(BPE) and Ir(ppy)_2_(TPP). (**a**) MO 161; (**b**) MO 160; (**c**) MO159; (**d**) MO 158; (**e**) MO157; (**f**) MO156; (**g**) MO117; (**h**) MO116; (**i**) MO 115; (**j**) MO 114; (**k**) MO 113; (**l**) MO 112.

For a clear understanding of the electronic transitions of Ir(ppy)_2_(BPE) and Ir(ppy)_2_(TPP), their first four singlet and triplet electronic transitions were calculated by the density functional theory (DFT) method. For both Ir complexes, it is observed from Figure 8 that their unoccupied frontier molecular orbitals (LUMO and LUMO+1) are mainly composed of the ppy ligand, with minor contribution from the BPE/TPP ligands and a slim contribution from the central Ir atom. Their occupied frontier molecular orbitals (HOMO, HOMO-1, HOMO-2 and HOMO-3) consist of the Ir atom and ppy ligand, admixed with some contribution from the BPE ligand. The contribution from auxiliary ligands (BPE or TPP) to these frontier molecular orbitals is not dominant, as shown in Table 3 and Table 4. Since the first four singlet and triplet electronic transitions correspond to excitations between these frontier molecular orbitals, these transitions were assigned as a characteristic of MLLCT. In addition, it is rational to assume that the electronic transition of Ir(ppy)_2_(L^X) complexes is controlled by the Ir atom and ppy ligand. This result explains why the photophysical performance of Ir(C^N)_3_ and Ir(C^N)_2_(L^X) complexes is dominated and affected by the major ligand C^N, while the auxiliary ligand L^X exerts only a moderate effect on photophysical performance [4,5].

#### 3.3.3. Confirm the Potential Surface Crossing of the ^3^MLLCT Excited State via Temperature-Dependent Emission Spectra

Finally, the potential surface crossing and energy transfer from ^3^ML_BPE_CT/^3^ML_BPE_L_ppy_CT to ^3^ML_ppy_CT should be confirmed before we come to a conclusion that the BPE ligand offers large steric hindrance to decrease the ACQ effect, allowing efficient emissive decay of the ^3^ML_ppy_CT excited state. This is because the ^3^ML_BPE_CT and ^3^ML_BPE_L_ppy_CT excited states have higher energy, which means that their electrons may interact with the surrounding environment, leading to the ACQ effect.

**Figure 9:**
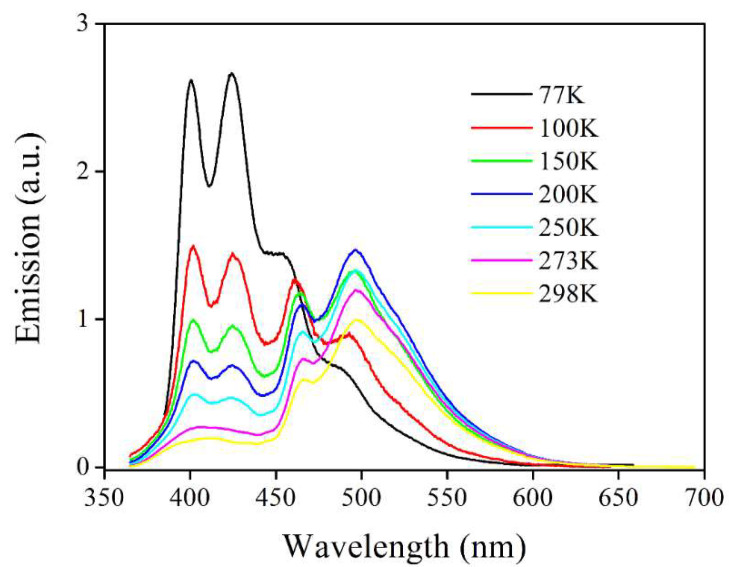
Temperature-dependent emission spectra of Ir(ppy)_2_(BPE) upon decreasing the temperature from 298 K to 77 K.

^3^ML_ppy_CT is the lowest excited state and thus the most stable excited state waiting for emissive decay. In addition, the excited electrons localizing at the ppy ligand may be protected by the BPE ligand and its steric hindrance, decreasing the ACQ effect. To confirm this statement, temperature-dependent emission spectra of Ir(ppy)_2_(BPE) upon decreasing the temperature from 298 K to 77 K were recorded, and these are shown as Figure 9. It is observed that ^3^ML_BPE_CT and ^3^ML_BPE_L_ppy_CT emission bands obviously increased with a decreasing temperature. Particularly, the ^3^ML_BPE_CT emission band was enhanced by ~21 times at 77 K compared to that at 298 K. Clearly, the potential surface crossing was limited by the low temperature, and the energy transfer from ^3^ML_BPE_CT/^3^ML_BPE_L_ppy_CT to ^3^ML_ppy_CT greatly decreased, leading to enhanced emission from ^3^ML_BPE_CT and ^3^ML_BPE_L_ppy_CT. With this energy transfer procedure confirmed, it is now safe to conclude that the ACQ effect was efficiently decreased by the auxiliary BPE ligand, showing efficient green emission in the solid state.

## 4. Conclusions

To summarize, two phosphorescent Ir complexes with a decreased ACQ effect in the solid state were synthesized using BPE and TPP as auxiliary ligands. Their successful synthesis was confirmed by their single crystals. A distorted octahedral coordination mode was observed owing to the heterogeneous ligands. The large steric hindrance in these two ligands minimized the π-π interaction between complex molecules; therefore, the AIPE influence was minimized, allowing enough time for the transformation of the highly phosphorescent ^3^MLLCT (metal-to-ligand-ligand charge transfer) excited state. A detailed photophysical comparison between these Ir complexes in solution, in the solid state, and in electrospun fibers was performed to confirm the realization of limited aggregation-caused quenching (ACQ). The potential surface crossing and energy transfer from ^3^ML_BPE_CT/^3^ML_BPE_L_ppy_CT to ^3^ML_ppy_CT in these Ir complexes was revealed by density functional theory calculation and temperature-dependent emission. It was confirmed that these two phosphorous ligands offered large steric hindrance, which decreased the ACQ effect, allowing the efficient emissive decay of the ^3^ML_ppy_CT excited state. This is one of the several luminescent Ir complexes with a high emission yield (Φ = 0.27) and long emission lifetime of 0.43 μs in the solid state. This finding shall be useful for the future design of phosphorescent Ir complexes that aim to decrease the ACQ effect.

## Figures and Tables

**Table 1:** Selected geometric parameters of Ir(ppy)_2_(BPE) and Ir(ppy)_2_(TPP).

Ir(ppy_)2_(BPE)				Ir(ppy)_2_(TPP)			
Bond Length	(Å)	Bond Angle	(°)	Bond Length	(Å)	Bond Angle	(°)
Ir-N1	2.08	N1-Ir-C1	79.56	Ir-N1	2.07	N1-Ir-C1	80.59
Ir-C1	2.05	N2-Ir-C2	79.39	Ir-C1	2.03	N2-Ir-C2	79.71
Ir-N2	2.09	P1-Ir-P2	99.22	Ir-N2	2.07	Cl1-Ir-P1	90.77
Ir-C2	2.04	N1-Ir-P1	98.63	Ir-C2	2.03	N1-Ir-P1	97.41
Ir-P1	2.48	N1-Ir-P2	92.99	Ir-P1	2.48	N1-Ir-C2	86.64
Ir-P2	2.54	N2-Ir-P1	88.61	Ir-Cl1	2.50	N2-Ir-Cl1	90.43
Ir…O	3.49	N2-Ir-P2	98.22	Ir…O	N/A	N2-Ir-P1	99.88
cell-a	11.85	cell-α	90.00	cell-a	9.88	cell-α	90.00
cell-b	14.25	cell-β	90.00	cell-b	14.94	cell-β	90.00
cell-c	31.21	cell-γ	93.58	cell-c	22.58	cell-γ	100.87

**Table 2:** Selected photophysical parameters of Ir(ppy)_2_(BPE) and Ir(ppy)_2_(TPP) in the solution (1 μM), solid state, and PVP fiber.

	Ir(ppy)_2_(BPE)						Ir(ppy)_2_(TPP)					
Status	λ_abs_ (nm)	λ_edg_ (nm)	λ_em_ (nm)	τ (μs)	Φ	k_r_/k_nr_ (×105 s^−1^)	λ_abs_ (nm)	λ_edg_ (nm)	λ_em_ (nm)	τ (μs)	Φ	k_r_/k_nr_ (×105 s^−1^)
solution	239, 319, 369	404	411, 460, 506, 525	τ1 = 0.39	0.43	7.54/10.01	237, 318, 367	409	488	τ1 = 0.02	0.01	0.345/34.14
				τ2 = 0.53						τ2 = 0.24		
				τ3 = 0.66						τ3 = 0.44		
				τ = 0.57						τ = 0.29		
solid	a	a	410, 466, 496	τ1 = 0.39	0.27	6.28/16.98	a	a	b	b	b	N/A
				τ2 = 0.41								
				τ3 = 0.58								
				τ = 0.43								
PVP, 5%	216, 257, 311,433	510	408, 458, 488	0.71	0.52	7.32/6.76	260, 310, ~425	512	448, 482, 508	0.33	0.04	1.21/29.09
PVP, 10%	215, 258, 310, 433	511	409, 460, 489	0.87	0.55	6.32/5.17	260, 310, ~425	513	451, 485, 508	0.61	0.08	1.31/15.08
PVP, 15%	217, 258, 312, 435	511	409, 461, 491	0.83	0.53	6.38/5.66	261, 310, ~425	513	~452, 485, 509	0.66	0.08	1.21/13.94

a = solid samples were too thick for light to penetrate, no valid absorption signal was recorded. b = no valid emission signal was recorded.

**Table 3:** Ir(ppy)_2_(BPE) percentage composition of singlet/triplet transitions and related molecular orbitals calculated at the RB3LYP/LANL2DZ level.

MO/Transition	Composition (%)		
	Ir	ppy	BPE
161 (LUMO+1)	7.1	71.3	21.6
160 (LUMO)	5.2	77.7	13.5
159 (HOMO)	40.9	39.5	19.7
158 (HOMO-1)	10.7	69.8	18.1
157 (HOMO-2)	22.3	57.5	18.2
156 (HOMO-3)	14.3	63.1	22.0
S0 → S1	MLLCT	MO159 → 160 (95.3)	
S0 → S2	MLLCT	MO159 → 161 (96.6)	
S0 → S3	MLLCT	MO158 → 160 (92.3)	
S0 → S4	MLLCT	MO158 → 161 (87.6)	
S0 → T1	MLLCT	MO157 → 160 (35.3)/158 → 160 (23.3)/159 → 160 (18.1)	
S0 → T2	MLLCT	MO159 → 161 (41.3)/156 → 161 (16.5)/158 → 161 (15.3)	
S0 → T3	MLLCT	MO159 → 160 (54.5)/157 → 160 (26.1)	
S0 → T4	MLLCT	MO159 → 161 (47.7)/156 → 161 (26.1)	

**Table 4:** Ir(ppy)_2_(TPP) percentage composition of singlet/triplet transitions and related molecular orbitals calculated at the RB3LYP/LANL2DZ level.

MO/Transition	Composition (%)		
	Ir	ppy	TPP
117 (LUMO+1)	11.0	84.2	3.8
116 (LUMO)	4.7	85.9	9.7
115 (HOMO)	41.0	35.1	5.8
114 (HOMO-1)	43.4	27.3	12.8
113 (HOMO-2)	38.4	43.8	11.1
112 (HOMO-3)	8.8	44.1	25.3
S0 → S1	MLLCT	MO115 → 116 (91.1)	
S0 → S2	MLLCT	MO114 → 116 (87.1)/113 → 116 (9.9)	
S0 → S3	MLLCT	MO115 → 117 (95.9)	
S0 → S4	MLLCT	MO114 → 117 (65.4)/113 → 116 (27.7)	
S0 → T1	MLLCT	MO114 → 116 (35.3)/115 → 116 (35.9)	
S0 → T2	MLLCT	MO115 → 117 (58.6)/112 → 117 (9.0)	
S0 → T3	MLLCT	MO115 → 116 (50.2)/114 → 116 (37.9)	
S0 → T4	MLLCT	MO113 → 116 (85.9)	

## Data Availability

All data can be found from the manuscript.

## References

[1] Jo J., Lee H.Y., Liu W.J., Olasz A., Chen C.-H., Lee D. (2012). Reactivity-Based Detection of Copper(II) Ion in Water: Oxidative Cyclization of Azoaromatics as Fluorescence Turn-On Signaling Mechanism. J. Am. Chem. Soc..

[2] Duke R.M., Veale E.B., Pfeffer F.M., Kruger P.E., Gunnlaugsson T. (2010). Colorimetric and fluorescent anion sensors: An overview of recent developments in the use of 1,8-naphthalimide-based chemosensors. Chem. Soc. Rev..

[3] Boens N., Leen V., Dehaen W. (2012). Fluorescent indicators based on BODIPY. Chem. Soc. Rev..

[4] Ji S.M., Wu W.H., Wu W.T., Song P., Han K.L., Wang Z.G., Liu S.S., Guo H.M., Zhao J.Z. (2010). Tuning the luminescence lifetimes of ruthenium(ii) polypyridine complexes and its application in luminescent oxygen sensing. J. Mater. Chem..

[5] Yang X.D., Shen B.W., Jiang Y.N., Zhao Z.X., Wang C.X., Ma C., Yang B., Lin Q. (2013). A novel fluorescent polymer brushes film as a device for ultrasensitive detection of TNT. J. Mater. Chem. A.

[6] Hargrove A.E., Nieto S., Zhang T., Sessler J.L., Anslyn E.V. (2011). Artificial Receptors for the Recognition of Phosphorylated Molecules. Chem. Rev..

[7] McQuade D.T., Pullen A.E., Swager T.M. (2000). Conjugated Polymer-Based Chemical Sensors. Chem. Rev..

[8] Chan Y.-H., Chen J.X., Liu Q.S., Wark S.E., Son D.H., Batteas J.D. (2010). Ultrasensitive Copper(II) Detection Using Plasmon-Enhanced and Photo-Brightened Luminescence of CdSe Quantum Dots. Anal. Chem..

[9] Thomas K.R., Velusamy M., Lin J.T., Chien C.H., Tao Y.T., Wen Y.S., Hu Y.H., Chou P.T. (2005). Efficient red-emitting cyclometalated iridium (III) complexes containing lepidine-based ligands. Inorg. Chem..

[10] Zhang L., Li B., Shi L., Li W. (2009). Synthesis, structures, and photophysical properties of fluorine-functionalized yellow-emitting iridium complexes. Opt. Mater..

[11] Yang L., Okuda F., Kobayashi K., Nozaki K., Tanabe Y., Ishii Y., Haga M.A. (2008). Syntheses and phosphorescent properties of blue emissive iridium complexes with tridentate pyrazolyl ligands. Inorg. Chem..

[12] Constable E.C., Housecroft C.E., Schonhofer E., Schonle J., Zampese J.A. (2012). Softening the donor set for light-emitting electrochemical cells:[Ir (ppy) 2 (N^ N)]+,[Ir (ppy) 2 (P^ P)]+ and [Ir (ppy) 2 (P^ S)]+ salts. Polyhedron.

[13] Luo S.X., Wei L., Zhang X.H., Lim M.H., Lin K.X.V., Yeo M.H.V., Zhang W.H., Liu Z.P., Young D.J., Hor T.S.A. (2013). Enhanced Emission and Analyte Sensing by Cinchonine Iridium(III) Cyclometalated Complexes Bearing Bent Diphosphine Chelators. Organometallics.

[14] Martie D.R., Bansal A.K., Mascio V.D., Cordes D.B., Henwood A.F., Slawin A.M.Z., Kamer P.C.J., Martinez-Sarti L., Pertegas A., Bolink H.J. (2016). Enhancing the photoluminescence quantum yields of blue-emitting cationic iridium (III) complexes bearing bisphosphine ligands. Inorg. Chem. Front..

[15] Wang Y., Teng F., Tang A., Wang Y., Xu X. (2005). Chlorobis [2-(2-pyridyl) phenyl-κ2N, C1](triphenylphosphine-κP) iridium (III) dichloromethane sesquisolvate. Acta Cryst..

[16] Shen X., Wang F.L., Sun F., Zhao R., Wang X., Jing S., Xu Y., Zhu D.R. (2011). New 2-phenyl-5-nitropyridyl containing iridium (III) cyclometalated complexes: Syntheses, structures, electrochemistry and photophysical properties. Inorg. Chem. Commun..

[17] Kranenburg M., van der Brugt Y.E.M., Kamer P.C.J., van Leeuwen P.W.N.M. (1995). New diphosphine ligands based on heterocyclic aromatics inducing very high regioselectivity in rhodium-catalyzed hydroformylation: Effect of the bite angle. Organometallics.

[18] Li X.Y., Zhou Y.L., Zheng Z.Z., Yue X.L., Dai Z.F., Liu S.Q., Tang Z.Y. (2009). Glucose biosensor based on nanocomposite films of CdTe quantum dots and glucose oxidase. Langmuir.

[19] Wang X., Tian W., Liao M.Y., Bando Y., Golberg D. (2014). Recent advances in solution-processed inorganic nanofilm photodetectors. Chem. Soc. Rev..

[20] Grosso D. (2011). How to exploit the full potential of the dip-coating process to better control film formation. J. Mater. Chem..

[21] Liu C., Song X.L., Rao X.F., Xing Y., Wang Z.G., Zhao J.Z., Qiu J.S. (2014). Novel triphenylamine-based cyclometalated platinum (II) complexes for efficient luminescent oxygen sensing. Dye. Pigment.

[22] Guan W., Zhou W., Lu J., Lu C. (2015). Luminescent films for chemo- and biosensing. Chem. Soc. Rev..

